# Supplementary material for: Identification of Vinyl Sulfone Derivatives as EGFR Tyrosine Kinase Inhibitor: In Vitro and In Silico Studies
Source: Molecules. 2021 Apr 12;26(8):2211. doi: 10.3390/molecules26082211 (PMC8069501; doi:10.3390/molecules26082211)
Supplement: Supplementary file 1 [file molecules-26-02211-s001.pdf]

Supplementary Information

Identification of Vinyl Sulfone Derivatives as EGFR Tyrosine Kinase Inhibitor: In Vitro and In Silico Studies

Thitinan Aiebchun [1], Panupong Mahalapbutr [2], Atima Auepattanapong [3], Onnicha Khaikate [3], Supaphorn Seetaha [4], Lueacha Tabtimmai [5], Chutima Kuhakarn [3], Kiattawee Choowongkomon [4,*] and Thanyada Rungrotmongkol [1,6,*]

[1] Biocatalyst and Environmental Biotechnology Research Unit, Department of Biochemistry, Faculty of Science, Chulalongkorn University, Bangkok, Thailand; thitinan1906@gmail.com
[2] Department of Biochemistry, Faculty of Medicine, Khon Kaen University, Khon Kaen 40002, Thailand; panupma@kku.ac.th
[3] Department of Chemistry and Center of Excellence for Innovation in Chemistry (PERCH-CIC),
Faculty of Science, Mahidol University, Bangkok, Thailand; iceatima.12@gmail.com (A.A.); onnicha.khai@gmail.com (O.K.); chutima.kon@mahidol.ac.th (C.K.)
[4] Department of Biochemistry, Faculty of Science, Kasetsart University, Chatuchak, Bangkok 10900, Thailand; supaporn.se@ku.th
[5] Department of Biotechnology, Faculty of Applied Science, King Mongkut's University of Technology of North Bangkok, Bangkok, Thailand; Lueacha.t@sci.kmutnb.ac.th
[6] Program in Bioinformatics and Computational Biology, Faculty of Science, Chulalongkorn University, Bangkok, Thailand
* Corresponding: kiattawee.c@ku.th (K.C.); t.rungrotmongkol@gmail.com (T.R.); Tel: +66-2218-5426 (T.R.);
Fax: +66-2218-5418 (T.R.)

Table of Contents

1. **Figure S1.** Superimposition of ligands between X-ray structure and CDOCKER docking	S3
2. **Figure S2.** Binding pose between VF16 and known drug within EGFR-TK	S3
3. **Figure S3.** The IC_{50} curves of kinase inhibitory activity of VF16 against EGFR-TK, JAK3, and HER2	S4

1. Figure S1

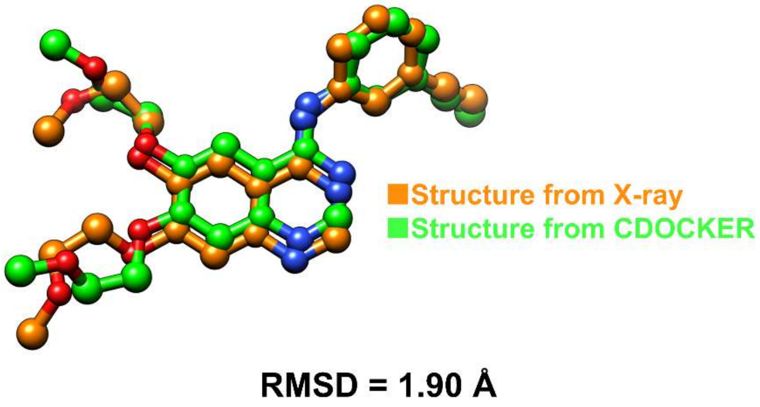

Figure S1. Superimposition of ligands between X-ray structure (orange) and CDOCKER docking (green).

2. Figure S2

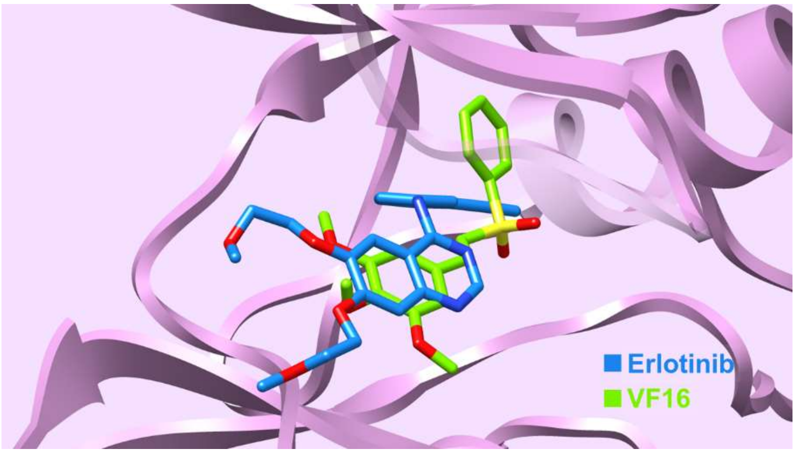

Figure S2. Binding pose between VF16 and known drug within EGFR-TK.

3. Figure S3

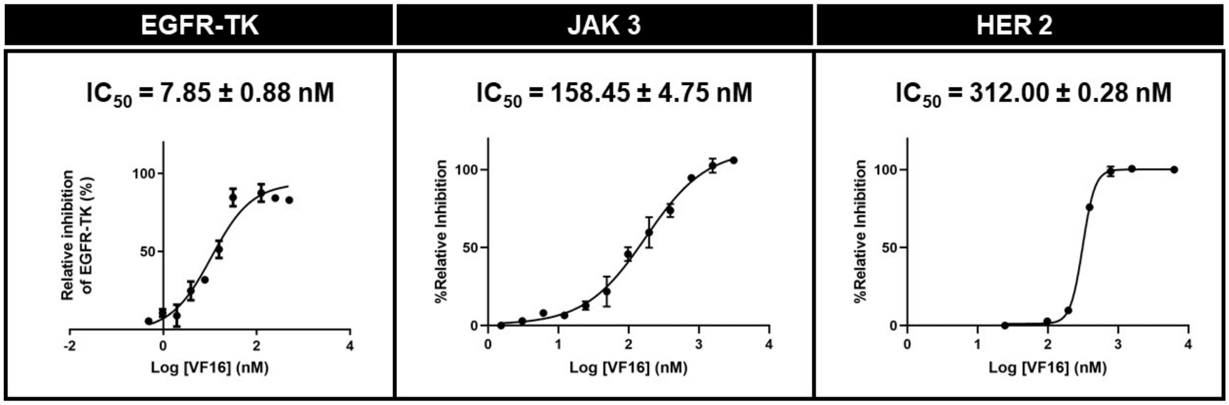

Figure S3. The IC_{50} curves of kinase inhibitory activity of VF16 against EGFR-TK, JAK3, and HER2.
